# Double-Barrier Memristive Devices for Unsupervised Learning and Pattern Recognition

**DOI:** 10.3389/fnins.2017.00091

**Published:** 2017-02-28

**Authors:** Mirko Hansen, Finn Zahari, Martin Ziegler, Hermann Kohlstedt

**Affiliations:** Nanoelektronik, Technische Fakultät, Christian-Albrechts-Universität zu KielKiel, Germany

**Keywords:** memristive devices, synaptic plasticity, spiking neuron, neural network, neuromorphic systems, unsupervised learning

## Abstract

The use of interface-based resistive switching devices for neuromorphic computing is investigated. In a combined experimental and numerical study, the important device parameters and their impact on a neuromorphic pattern recognition system are studied. The memristive cells consist of a layer sequence Al/Al_2_O_3_/Nb_*x*_O_*y*_/Au and are fabricated on a 4-inch wafer. The key functional ingredients of the devices are a 1.3 nm thick Al_2_O_3_ tunnel barrier and a 2.5 mm thick Nb_*x*_O_*y*_ memristive layer. Voltage pulse measurements are used to study the electrical conditions for the emulation of synaptic functionality of single cells for later use in a recognition system. The results are evaluated and modeled in the framework of the plasticity model of Ziegler et al. Based on this model, which is matched to experimental data from 84 individual devices, the network performance with regard to yield, reliability, and variability is investigated numerically. As the network model, a computing scheme for pattern recognition and unsupervised learning based on the work of Querlioz et al. (2011), Sheridan et al. (2014), Zahari et al. (2015) is employed. This is a two-layer feedforward network with a crossbar array of memristive devices, leaky integrate-and-fire output neurons including a winner-takes-all strategy, and a stochastic coding scheme for the input pattern. As input pattern, the full data set of digits from the MNIST database is used. The numerical investigation indicates that the experimentally obtained yield, reliability, and variability of the memristive cells are suitable for such a network. Furthermore, evidence is presented that their strong *I*–*V* non-linearity might avoid the need for selector devices in crossbar array structures.

## Introduction

The brains of humans, mammals, and even simple living species like invertebrates are well-adapted to permanently changing environments. Their nervous systems exhibit remarkable interactions with their surroundings—a result of millions of years of evolution and explicable by Darwinism (Shanahan, 2004). As a result, biological systems are presently unmatched in the efficient way in which they are able to perform cognitive tasks, such as pattern recognition, with extremely low power consumption. It is therefore no surprise that attempts have been made to develop bio-inspired computing systems, so-called neuromorphic systems, with the goal of reaching the performance and power efficiency of biological systems (Liu et al., 2002; Chicca et al., 2014). Machine learning dates back to the early days of serial, binary computation based on the von Neumann architecture, but today's artificial neural systems are still only partially able to mimic biological systems. The huge power dissipation, the long computational time, and the need for large datasets are among the major problems faced in attempting to realize artificial neural networks (ANNs).

In this context, analog very large-scale integration (VLSI) based on silicon complementary metal-oxide-semiconductor (CMOS) technology (Liu et al., 2002; Chicca et al., 2014) might provide advantages to software-dominated neuroinformatics (Amit, 1989; Würtz, 2008). This field has gained new momentum with the advent of the concept of memristive devices (memristors) (Strukov et al., 2008; Jo et al., 2010; Jeong et al., 2013). The memristor is a device whose resistance depends on its history of applied potentials. It was predicted in 1971 by Chua (1971). In the memristor model, the resistance (or memristance) *M*(*x, V, t*) of the device can be expressed by an internal state variable *x*(*t*), which depends on the applied voltage *V*(*t*) and on itself:

(1) $$\frac{dx}{dt}=f\left(x\left(t\right),V\left(t\right)\right).$$

Here, *f* describes the dynamics of the updating process of *x*(*t*). In many memristive devices, *f* describes the process of ion migration due to an externally applied voltage.

Recently, Hebbian learning as an important biological concept has been realized with single memristive devices by emulating spike-timing-dependent plasticity (STDP; Jo et al., 2010; Zamarreño-Ramos et al., 2011), long-term potentiation, and long-term depression (Ohno et al., 2011). These properties are important cellular mechanisms of memory and learning in neural networks (Ziegler et al., 2015).

Although the structure of memristive devices is rather simple (consisting of a capacitor-like metal-insulator-metal sequence in the simplest case), the underlying physical mechanism is often unclear. The function of many memristive devices is based on the presence of conductive filaments, which results in poor switching reproducibility and high inter-device variability and often requires an initial and individual electrical forming step (Szot et al., 2006; Waser et al., 2009; Ha and Ramanathan, 2011; Yang et al., 2013; Dirkmann et al., 2015). Furthermore, memristive devices have mainly been considered for application in future resistive random-access memories (RRAMs; Itoh et al., 2006), whose system architecture and functionality are similar to those of digital memories. Here, switching times in the nanosecond range, long data retention times (10 years), low device variability, and good fatigue performance are essential requirements. Memristive devices for use in neuromorphic systems are subject to different requirements, and other resistive switching concepts can be of interest (Ziegler et al., 2015), such as interface-based memristive devices.

In interface-based devices, uniform interface effects lead to homogeneous rather than abrupt changes in resistance. Furthermore, such devices do not suffer from the randomness generated by electroforming or filament growth (Kohlstedt et al., 1993; Baikalov et al., 2003; Meyer et al., 2008; Park et al., 2008; Sawa, 2008; Baik and Lim, 2010; Hu et al., 2011; Jeong et al., 2011; Aoki et al., 2014; Mikheev et al., 2014). In some designs for these devices, the resistive switching results from changes at a Schottky-like contact (Baikalov et al., 2003; Mikheev et al., 2014). In another approach, the electron tunneling probability is varied when a memristive layer is in contact with a tunnel barrier (Kohlstedt et al., 1993; Meyer et al., 2008; Baik and Lim, 2010; Jeong et al., 2011). Recently, we have been able to combine both concepts into a single device by sandwiching an ultra-thin memristive layer between a tunnel barrier and a Schottky-like contact (Hansen et al., 2015). This offers several benefits: the tunnel barrier defines the lower resistance boundary and limits the current through the device. Further, both the tunnel and the Schottky barrier define chemical barriers to ion migration, leading to improved data retention compared with single-barrier concepts. However, the possible benefits for bio-inspired neuromorphic circuits are not that obvious and hence have to be explored.

The purpose of this work is to provide a thorough analysis of interface-based memristive devices and their potential use in bio-inspired neuromorphic systems. With this aim in mind, double-barrier memristive devices with a layer sequence Al/Al_2_O_3_/Nb_*x*_O_*y*_/Au have been fabricated. The thickness of the Al_2_O_3_ tunnel barrier is ~1.3 nm and that of the memristive Nb_*x*_O_*y*_ layer is 2.5 nm. The use of an ultra-thin memristive layer allows the required electrical field strength for oxygen ion migration to be achieved, reducing the resistance variation due to interfacial processes, and also allows interference between electron tunneling and the Schottky barrier (Hansen et al., 2015). To determine the important device characteristics and necessary electrical conditions for use in neuromorphic systems, automated measurements are undertaken, providing valid statistical data from 84 single devices. The resistive switching properties are investigated using voltage pulses with different lengths and amplitudes. The yield, reliability, and device variability are determined. To obtain a realistic mathematical description of the device behavior from the obtained experimental data for network-level applications, the memristive plasticity model of Ziegler et al. (2015) is used. This model allows us to explore functional consequences of the use of individual memristive devices for the emulation of Hebbian plasticity. As guidelines for network simulation, previously reported neural-network-based paradigms are adopted (Querlioz et al., 2011, 2013; Sheridan et al., 2014; Zahari et al., 2015). In particular, a neural network for pattern recognition is simulated consisting of inhibitorily linked output neurons within a winner-takes-it-all architecture and a homeostasis-like rule for the spiking-neurons thresholds. The network is trained on the MNIST database. On the basis of this pattern recognition network, essential requirements for the development of neuromorphic circuits with interface-based memristive devices are discussed.

The paper is organized as follows: Section Materials and Methods describes the experimental techniques used and the fabrication and electrical characterization of the memristive devices. It also presents the phenomenological synaptic learning model that we use to model the experimental data. The section closes with a brief description of the network architecture used for the pattern recognition simulation. Section Results contains the results of our investigation, namely, plasticity measurements, and network performance. The behavior of the fabricated devices is discussed with respect to their reliability and variability in Section Discussion.

## Materials and methods

### Electrical measurements

All measurements were performed using an Agilent E5260 source measurement unit. Current–voltage measurements (*I*–*V* curves) were obtained by sweeping the applied voltage and measuring the current simultaneously. For synaptic plasticity measurements on single devices, rectangular voltage pulses with different amplitudes, polarities, and pulse durations were applied to the devices. Positive voltage is defined as positive voltage on the top electrode and ground on the bottom electrode.

### Device fabrication and characterization

**Figure 1:**
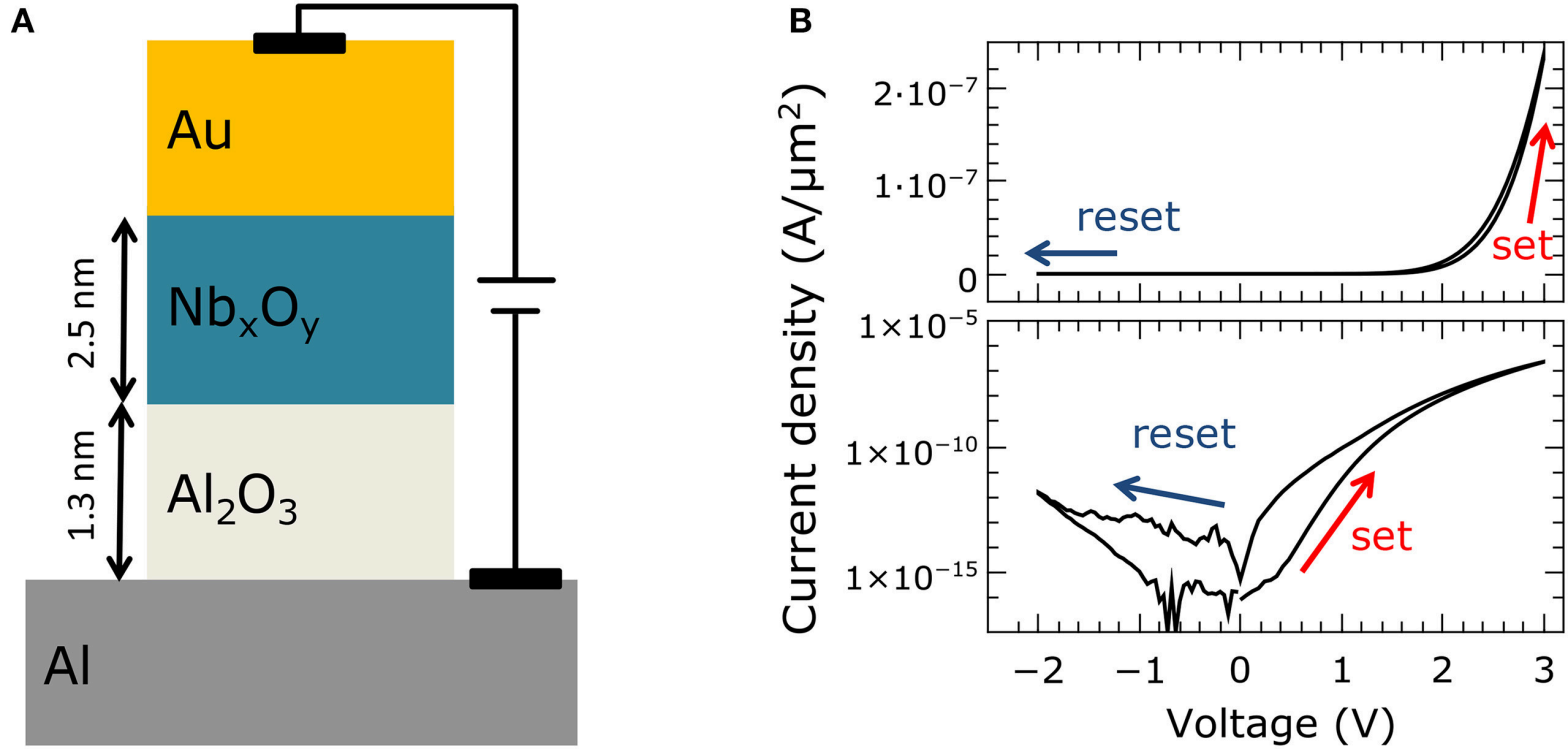
**Double-barrier memristive device: (A)** Schematic cross-section of the Al/Al_2_O_3_/Nb_*x*_O_*y*_/Au double-barrier memristive device. **(B)** Current density *J* as function of the applied bias voltage. In the upper graph in a linear scale for *J* was used, while in the lower panel the absolute value and a logarithmic scale was used to better visualize the obtained change in resistance.

The memristive devices were fabricated on 4-inch Si wafers with 400 nm SiO_2_ (thermally oxidized) using a standard optical lithography process (a schematic of the material stack is shown in Figure 1A). The junctions were arranged in 1 × 1 mm cells across the wafer, each containing six different contact sizes ranging from 70 to 2300 μm^2^. The devices were fabricated using the following procedure: First, the multilayer (including top and bottom electrodes) was deposited using DC magnetron sputtering, without breaking the vacuum. The Al_2_O_3_ tunnel barrier was fabricated by depositing Al, which was afterwards partially oxidized in situ. The Nb_*x*_O_*y*_ layer was deposited by reactive sputtering in an O_2_/Ar atmosphere. Following the subsequent lift-off, the junction area was defined by wet chemical etching of the Au top electrode using a potassium iodide solution. The etched parts were then covered with thermally evaporated SiO to insulate the bottom electrode from the subsequently deposited Nb wiring to contact the top electrode.

Figure 1B shows a typical recorded *I–V* curve of a double-barrier memristive device, exhibiting the typical pinched bipolar hysteresis of a memristive device (to reveal the *I–V* non-linearity more clearly, in the lower panel, the curve is plotted with a logarithmic scale on the vertical axis). The applied voltage was varied between −2 and 2.8 V, while the current was measured simultaneously. The voltage was ramped from 0 to 2.8 V to switch the device from its initial high-resistance state (HRS) to the low-resistance state (LRS). To reset the device, the voltage was decreased to −2 V. The asymmetry between positive and negative current can be attributed to the Schottky-like Nb_*x*_O_*y*_/Au contact, while the gradual resistance change indicates a non-filamentary resistance switching mechanism. This results from homogeneously changed interface properties (Hansen et al., 2015; Dirkmann et al., 2016). Neither an initial forming procedure nor current compliance was used. This is important for the integration of such devices into crossbar architectures, as we will discuss below.

The underlying physical mechanism of the resistive switching process has recently been studied in a combined experimental and theoretical investigation (Dirkmann et al., 2016). This investigation identified the transport of oxygen ions within the Nb_*x*_O_*y*_ as the key mechanism for the resistive switching process. During the set process, oxygen ions (within the Nb_*x*_O_*y*_) move under positive voltage toward the Au interface and affect essential interfacial parameters (e.g., the density of states, the local barrier height, and the barrier thickness) at the Al_2_O_3_/Nb_*x*_O_*y*_ and Nb_*x*_O_*y*_/Au (Schottky) interfaces simultaneously. By applying a negative bias voltage, the original ion distribution is restored. As a consequence, the electron transport is altered in accordance with the local ion distribution. This leads to the observed memristive *I*–*V* curve. For further information about the device performance and technology, the reader is referred to Hansen et al. (2015).

### Simulation model

To investigate device performance at the network level, we used a computing scheme for pattern recognition similar to Querlioz et al. (2011, 2013), Sheridan et al. (2014), Zahari et al. (2015). As a numerical model of the resistive switching under voltage pulsing, we used the plasticity model of Ziegler et al. (2015). This model is compatible with advanced biophysical plasticity models that account for experimental data on STDP. Furthermore, the model is suitable for describing plasticity emulation using memristive devices. We should mention here that the plasticity model provides a behavioral description of the memristive device, rather than an explanation of the underlying physical mechanisms. In the following, the plasticity model and network structure for pattern recognition are presented.

#### Plasticity model

The resistive switching of double-barrier memristive devices in neural circuits are described in the framework of the phenomenological learning model of Ziegler et al. (2015). This model links the change in conductance in a memristive device and the applied voltage pulse. The limited weight growth and the weight-dependent (memristive) learning rate make this model appropriate for memristive device and synapse emulations. In the plasticity model, the updating process for the synaptic weights ω is given by

(2) $$\frac{d\omega }{dt}=\beta (\omega )\omega (t)\left(1-\frac{1}{\omega_{\max}}\omega (t)\right),$$

where β is the weight-dependent learning rate and ω_max_ is the maximum synaptic weight. The resistive switching dynamic of the memristive device is manifested in β, which depends on the current device conductance and electrical stimuli. In particular, β depends on the switching mechanism of the memristive device and can lead to very different learning behaviors (Querlioz et al., 2015; Ziegler et al., 2015). In determining β for the double-barrier memristive device, two particularly important points should be noted: First, it is necessary to use distinct learning rates for potentiation (β_*p*_) and depression (β_*d*_) owing to the asymmetry between positive and negative voltages in the memristive device (cf. Figure 1B). Second, the synaptic weight ω is associated with the conductance *G* of a memristive device, which depends on height ΔV, length Δt, and number *n* of applied electrical pulses. Hence, we assume that β_*p*_ and β_*d*_ depend on ΔV, Δt, and *n*, so that

(3) $$\begin{array}{l} \beta_{p}(G,n,\Delta t,\Delta V)=k_{p}\;\alpha (\Delta V)\;\lambda (\Delta t)\;\left(1-\gamma G(n-1)\right)\\ \beta_{d}(G,n,\Delta t,\Delta V)=-k_{d}\;\alpha (\Delta V)\;\lambda (\Delta t)\;G(n-1). \end{array}$$

Here *k*_*p*_, *k*_*d*_, and γ are positive constants, while α and λ depend on the height ΔV and length Δt of the applied voltage stimulus. The functions α and λ account for the nonlinearity of the switching dynamics of the memristive device and have to be determined for the particular memristive device under consideration (see Section Results).

#### Network architecture for pattern recognition

**Figure 2:**
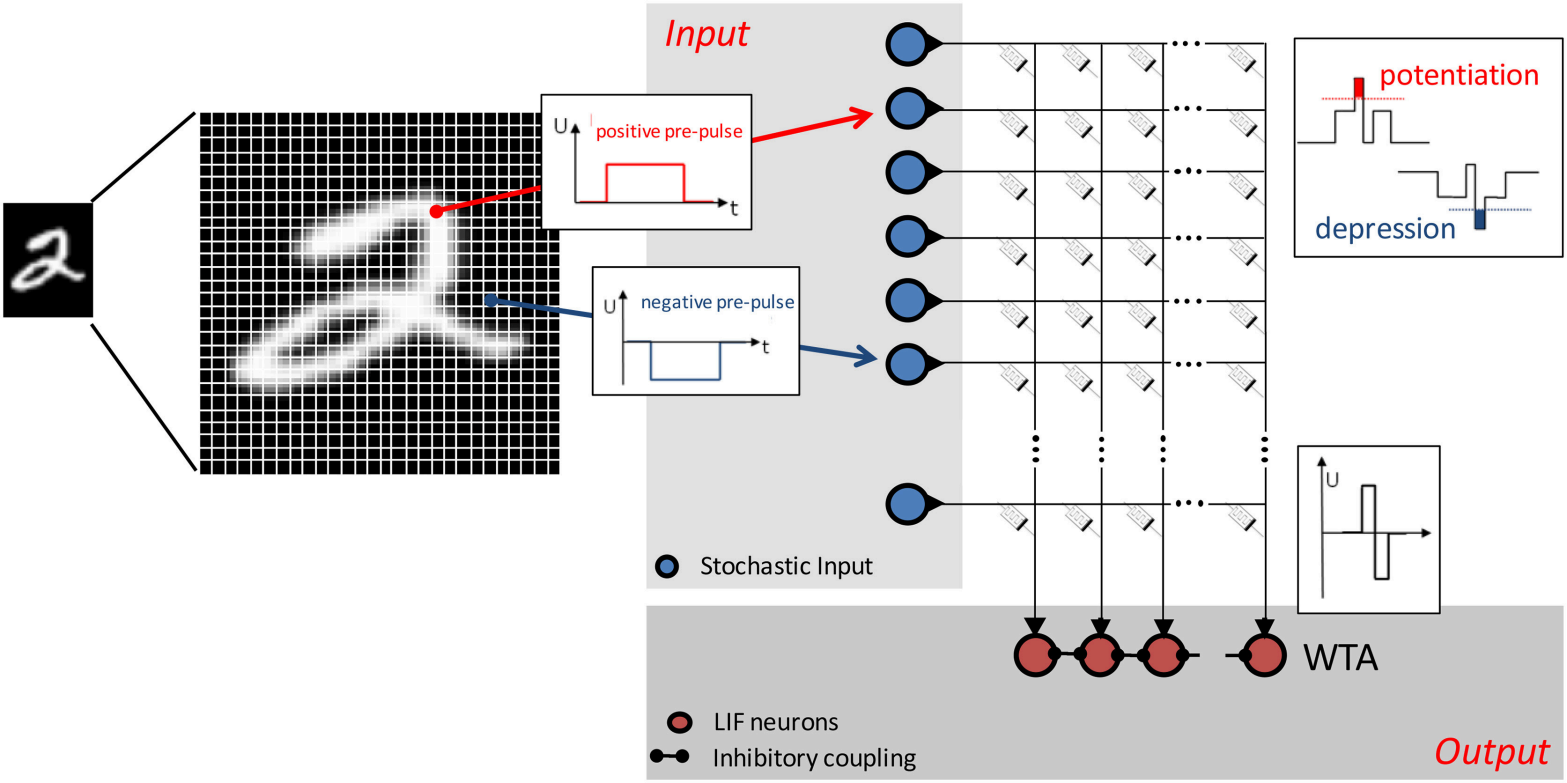
**Schematic of the simulated neural network: Positive and negative voltage pulses are applied to the input of the network, representing the intensity of the individual values of the 28 × 28 pixel MNIST image**. Stochastic coding of the input data has been implemented by Poisson-spiking input neurons (blue circles) with firing rates proportional to the intensity of the corresponding pixel of the input pattern. Red circles are leaky integrate-and-fire output neurons (LIF), which are laterally coupled in an inhibitory winner-takes-it-all network (WTA). The individual memristive devices are arranged in a crossbar structure. A local STDP-based learning rule has been implemented using the defined I–V nonlinearity of the memristive devices: Only the overlap (association) of pre- pulses and post-pulses leads to an increase (potentiation) or decrease (depression) of the device conductance.

The network structure is a two-layer feedforward network and is shown schematically in Figure 2. The memristive devices are arranged in a crossbar array to which input (blue circles) and output (red circles) neurons are connected. The individual pixels of the input pattern are coded by voltage pulses within the input layer. Leaky integrate-and-fire neurons are used in the output layer. These neurons are laterally coupled within an inhibitory winner-takes-it-all network including adaptive thresholds for the spiking, as proposed in Querlioz et al. (2011). The network can be briefly described as follows: The output neuron's spiking depends on the applied input pattern and the particular resistance of the memristive devices. The conductances of the memristive devices are changed by associative learning, and the voltage across each device depends on the input pattern and the activity of the particular output neuron. This enables unsupervised learning, because every output neuron creates its own specific receptive field during learning. Afterwards, in the recognition phase, each of the output neurons will spike in accordance with the previously learned pattern for a varying input.

The input patterns were taken from the MNIST database, which consists of 60,000 handwritten digits from 250 different writers. Each digit is stored in a 256-level grayscale image with 28 × 28 pixels (LeCun et al., 1998). In the network implemented here, the images are rearranged into a 784-row input vector applied to the input neurons (one neuron per image pixel). The value of each pixel is applied as a voltage pulse, which, in combination with the output neuron's pulse, causes a change in the conductance of a particular memristive cell in the cross-bar array. The device conductance is most strongly affected when the input and output pulses match, as sketched in Figure 2. In particular, the overlap of a negative input pulse with an output neuron's pulse leads to a decrease in the particular device conductance, while the combination of a positive input pulse with an output pulse increases the conductance of the memristive devices connected to the specific neurons. Memristive devices in input rows and output columns where there is no overlap of voltage pulses remain unaffected.

It is important for the operation of the network that the input data is stochastically coded (Querlioz et al., 2011, 2013; Sheridan et al., 2014; Zahari et al., 2015), and this has been achieved by the following steps: The image pixels are normalized to the interval −1 to 1. Following Sheridan et al. (2014), the grayscale value *p*_*i*_ of each pixel *i* is best normalized by

(4) $$p_{i}^{\text{norm}}=\frac{p_{i}-p_{\text{mean}}}{\max_{0\leq j\leq 784}\left(p_{j}-p_{\text{mean}}\right)},$$

where *p*_mean_ is the mean grayscale value of all pixels for an individual image. The absolute value of $p_{i}^{\text{norm}}$ denotes the probability of an input spike generation. Therefore, a random number *r* for each pixel is generated at each iteration step. If the condition $r\leq \;|p_{i}^{\text{norm}}|$ is satisfied, an input pulse is generated. The sign of $p_{i}^{\text{norm}}$ corresponds to the polarity of the input voltage (see Figure 2).

Leaky integrate-and-fire neurons are used as output neurons and are arranged in a laterally coupled inhibitory network (see Figure 2). To guarantee that individual input patterns are learned by different output neurons, the winner-takes-it-all approach is used, in which the first spiking neuron resets the integration of all other neurons. This allows unsupervised learning with the network structure that is used. Crucial for unsupervised learning is an adjustable neuron firing threshold, which guarantees that all output neurons participate equivalently in the learning phase. This can be motivated by considering the process of homeostasis in biological systems (Querlioz et al., 2011). Therefore, the threshold of a neuron to fire is increased whenever the spike number (activity) of a neuron is above the desired activity, and vice versa. Following Querlioz et al. (2011), this can be achieved by using

(5) $$\frac{dV_{\text{th}}}{dt}=\gamma_{\text{th}}(A_{\text{avg}}-A_{\text{tar}})$$

for the threshold voltage adaptation. Here, γ_th_, *A*_avg_, and *A*_tar_ are respectively the gain factor, the mean activity of an individual neuron, and the target activity (γ_th_ = 7.5 × 10^−3^ and *A*_tar_ = 10).

## Results

### Synaptic plasticity measurements

The basis for the emulation of synaptic functionalities with memristive devices is the fact that the synaptic weight ω between individual neurons can be related to the state variable *x* of a memristive device according to Equation (1). Zamarreño-Ramos et al. showed that the conductance of a memristive device is proportional to ω in the ideal voltage-driven memristor model (see Equation 1) (Zamarreño-Ramos et al., 2011). Hence, biological plasticity mechanisms can be emulated by changes in the device conductance under suitable voltage pulses. To study the capability of synaptic plasticity emulations using the Al/Al_2_O_3_/Nb_x_O_y_/Au double-barrier memristive device of Figure 1, voltage pulses with different pulse widths Δ*t* and amplitudes Δ*V* were applied to 86 individual devices.

**Figure 3:**
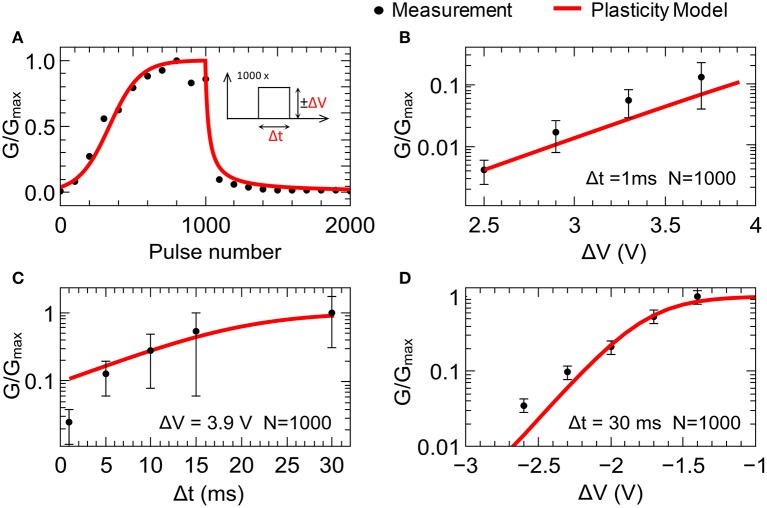
**Synaptic plasticity emulation: (A)** Typical plasticity measurement for 1000 potentiation pulses of +3.9 V and 1000 depression pulses of −2.5 V with pulse lengths of 1 ms. Each point represents the conductance after 100 pulses. In **(B–D)**, the change in device conductance after 1000 set (reset) voltage pulses with varying pulse amplitudes **(B,C)** and lengths Δt **(D**) is shown. Black dots reflect experimental data measured at 0.48 V. Red lines correspond to the data obtained using the plasticity model (model parameters are summarized in Table 1). The error bars reflect the device variability obtained from 86 individual devices. *G*_max_ is the averaged conductance of 86 devices after 1000 voltage pulses of 3.9 V in amplitude and 1 ms in length.

Figure 3A shows a typical plasticity measurement (conductance vs. pulse number). We found that the double-barrier memristive device exhibits a gradually changing conductance under voltage pulsing, as is desired for plasticity emulation. To study the pulse dependence, 1000 equivalent positive voltage pulses (potentiation pulses) followed by 1000 equivalent negative voltage pulses (depression pulses) were used, as shown schematically in the inset of Figure 3A. For the emulation of potentiation, voltage pulses of Δ*V* = 3.9 V and Δ*t* = 1 ms in length were chosen, while for depression the voltage pulse height was reduced to Δ*V* = −2.5 V. To measure the device conductance, voltage sweeps below the threshold voltage of the device up to 0.48 V were applied and the current was measured after every 100 potentiation/depression pulses. For better illustration, the obtained conductances *G*(*n*) were normalized by the average maximum conductance *G*_*max*_ (100 nS).

In order to study in greater detail the variations of the device conductance as the amplitude and length of the applied voltage pulses are changed, potentiation pulses with amplitudes ranging from 2.4 to 3.7 V and pulse lengths ranging from 1 to 30 ms were applied. The results are shown in Figures 3B,C. For the variation with amplitude, the pulse length was fixed at 1 ms, while for the variation with pulse length, the amplitude was fixed at 3.9 V. The data points reflect the device conductance after 1000 voltage pulses. For an amplitude of 2.4 V, the conductance only increases by 0.1–0.5% from the initial value *G*_0_. However, if amplitude is set to 3.7 V, there is a 10-fold increase (see Figure 3B). Of further interest is that the increase in conductance saturates for pulses above 10 ms (see Figure 3C). This is in accordance with the diffusion times of oxygen ions within the memristive layer, which are at the heart of the resistance switching process (Dirkmann et al., 2016).

The recorded data for synaptic depression are shown in Figure 3D. A voltage train of 1000 single voltage pulses of 30 ms length with amplitudes ranging from −1.4 to −2.6 V were applied to memristive cells that had previously been set to the high-conductance (low-resistance) state. The unchanged conductance for voltages above −1.4 V shows the threshold behavior for the reset process. In contrast, pulses with amplitudes of −2.6 V nearly reset the device conductance completely (see Figure 3D).

To adapt the experimental findings for the plasticity model described above, the functions α(Δ*V*) and λ(Δ*t*) from Equation (3) have to be determined. The functions α(Δ*V*) and λ(Δ*t*) account for the nonlinear dependencies of the learning rates β_*p*_ and β_*d*_ on the pulse amplitude and length. Good agreement with the experimental data (the black points in Figure 3) could be achieved using the following expressions (the red lines in Figure 3):

(6) $$\alpha (\Delta \text{ V})\;=\{\begin{array}{cc} \alpha_{1}\;\Delta V & \;for\;\Delta V>0,\;\\ \exp\left[\alpha_{2}\left(\Delta V-\alpha_{3}\right)\right] & for\;\Delta V<0, \end{array}$$

(7) $$\lambda \left(\Delta t\right)=\lambda_{1}\frac{1}{\Delta t}+\lambda_{2}\left(\Delta t-\lambda_{3}\right).$$

Here α_1, 2, 3_ and λ_1.2.3_ are positive constants, which are listed in Table 1 together with the other parameters of the fitting procedure. Owing to the differences between potentiation and depression, two different definitions for α(Δ*V*) are necessary: for potentiation, a linear dependence on Δ*V* is in good agreement with experiment, while an exponential function of Δ*V* is necessary to reproduce the experimentally recorded data for the emulation of synaptic depression. A linear function is best at reproducing the pulse length dependence (see Equation 7).

**Table 1:** **Parameters for the plasticity model**.

**Parameter**	**Value**
*k_*p, d*_*	5
α_1_	0.528 V^−1^
α_2_	1.75 V^−1^
α_3_	3.15 V
λ_1_	1 s
λ_2_	0.6 ms
λ_3_	1 s
γ	0.45

### Pattern recognition

**Figure 4:**
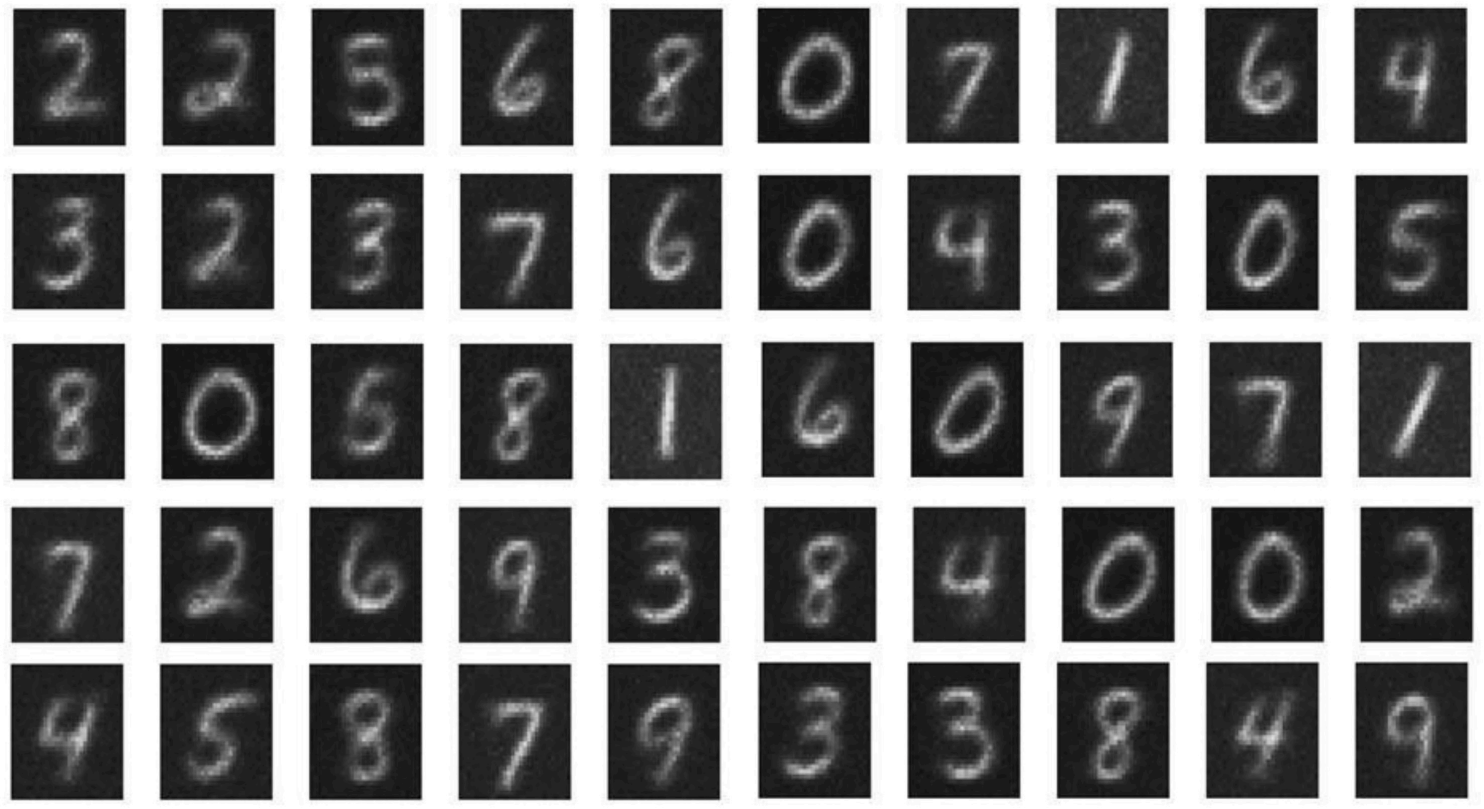
**Receptive fields: Obtained receptive fields after unsupervised learning in the case of 50 output neurons**. The white pixels correspond to the maximum conductance (strong synaptic weight), while the black pixels represent minimum conductance values (weak synaptic weight) of the memristive devices.

The network performance of the double-barrier memristive device was investigated by training the pattern recognition network with the complete MNIST dataset of 60,000 handwritten digits. To improve the learning performance of the network, the complete training set was applied three times to the network. After training, the network was tested by using the MNIST test dataset, which contains 10,000 digits that are not included in the training set. The performance of the network can be evaluated by counting the number of correctly identified digits. The recognition rate thereby obtained increases with the number of output neurons used. For 10, 20, 50, and 100 output neurons, recognition rates of 65, 70, 77, and 82%, respectively, were obtained. These rates are in good agreement with previously published investigations (Querlioz et al., 2011, 2013; Sheridan et al., 2014; Zahari et al., 2015). For visualization, each 784-value vector, above the particular output neuron, is rearranged into a 28 × 28 pixel image representing the receptive fields. A typical set of receptive fields for a simulation with 50 output neurons is shown in Figure 4. We found that the implemented network structure is able to learn all of the 10 input digits and the different details of each digit. We should mention here that the obtained recognition rates are much lower than those from other spiking network architectures (for an overview of different network architectures, the reader is referred to Diehl and Cook, 2015). However, the aim of our investigation was to study network requirements for the use of memristive devices in neural networks rather than to improve pattern recognition computing schemes.

**Figure 5:**
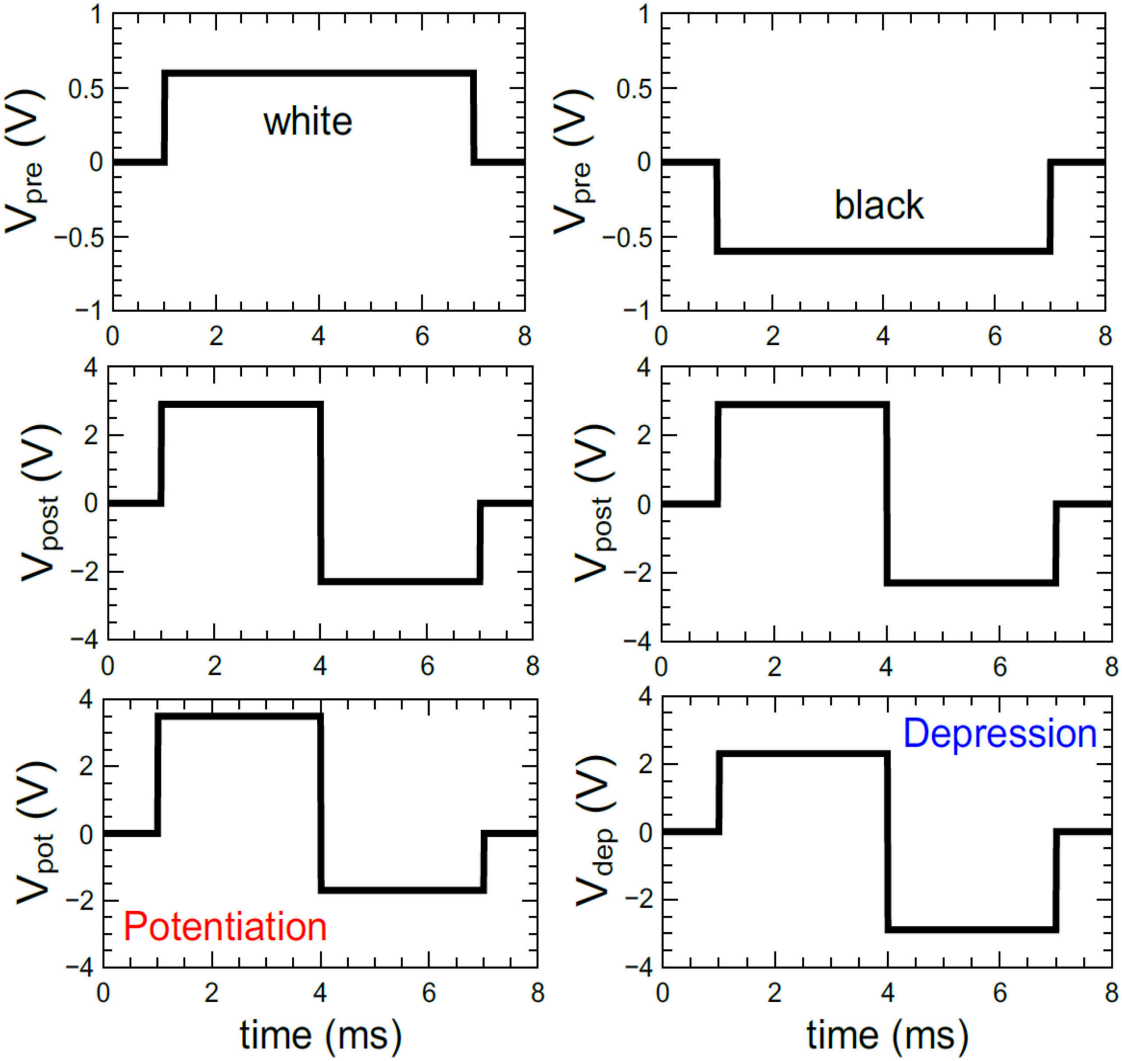
**Pulse forms used for the simulations: For the coding of the grayscale input images, positive and negative voltage pulses (*V*_*pre*_) with ±0.6 V in amplitude and 6 ms in width have been used**. The voltage pulses of the output neurons (*V*_*post*_) were set to a combination of a +2.9 V and a –2.3 V voltage pulses with a total duration of 6 ms. The combination of both voltage pulses (i.e., *V*_pre_ and *V*_post_) leads either to a potentiation (*V*_pot_) or depression (*V*_dep_) of the memristive device.

Appropriately chosen voltage pulses with lengths Δ*t* and amplitudes Δ*V* are important for the performance of the neural network, to precisely adjust the conductance of the double barrier memristive devices. In the case of matching input (*V*_pre_) and output (*V*_post_) pulses, the device conductance must be always affected. The voltage pulses used here are depicted in Figure 5. Based on the experimental data of Figure 3, suitable amplitudes and lengths of the voltage pulses were chosen for the simulations. For the input neuron, the pulse height *V*_pre_ was set to either +0.6 or −0.6 V (see the top row of Figure 5). The voltage pulses *V*_post_ generated by the leaky integrate-and-fire output neurons contain a positive part of 2.9 V followed by a negative part of −2.3 V (see the middle row of Figure 5). The combination of *V*_post_ with a negative input pulse (*V*_sum_ = −2.9 V) decreases the conductance, while the overlap of *V*_post_ with a positive input pulse (*V*_sum_ = +3.5 V) increases the conductance of the device connected to the specific neurons (see the bottom row in Figure 5). An important aspect of the double-barrier memristive device is its strong *I*–*V* nonlinearity (see Figure 1B). According to Figure 3, an increase in pulse height from 3 to 3.8 V leads to an increase in device conductance by a factor of 10, while below 1 V the device conductance remains unaffected. This provides a threshold voltage for the change in conductance, which is a very important requirement for the learning behavior of this crossbar-based computing scheme. Without this threshold voltage, the voltage drop across neighboring devices during the set or reset could be sufficient to change also the conductance of these neighboring devices. This problem is commonplace with devices that have a symmetrical *I*–*V* curve, requiring selector devices that access individual devices. Owing to the strong asymmetry and nonlinearity in the current of double-barrier memristive devices, there is no need for selector devices (Kügeler et al., 2009).

## Discussion

**Figure 6:**
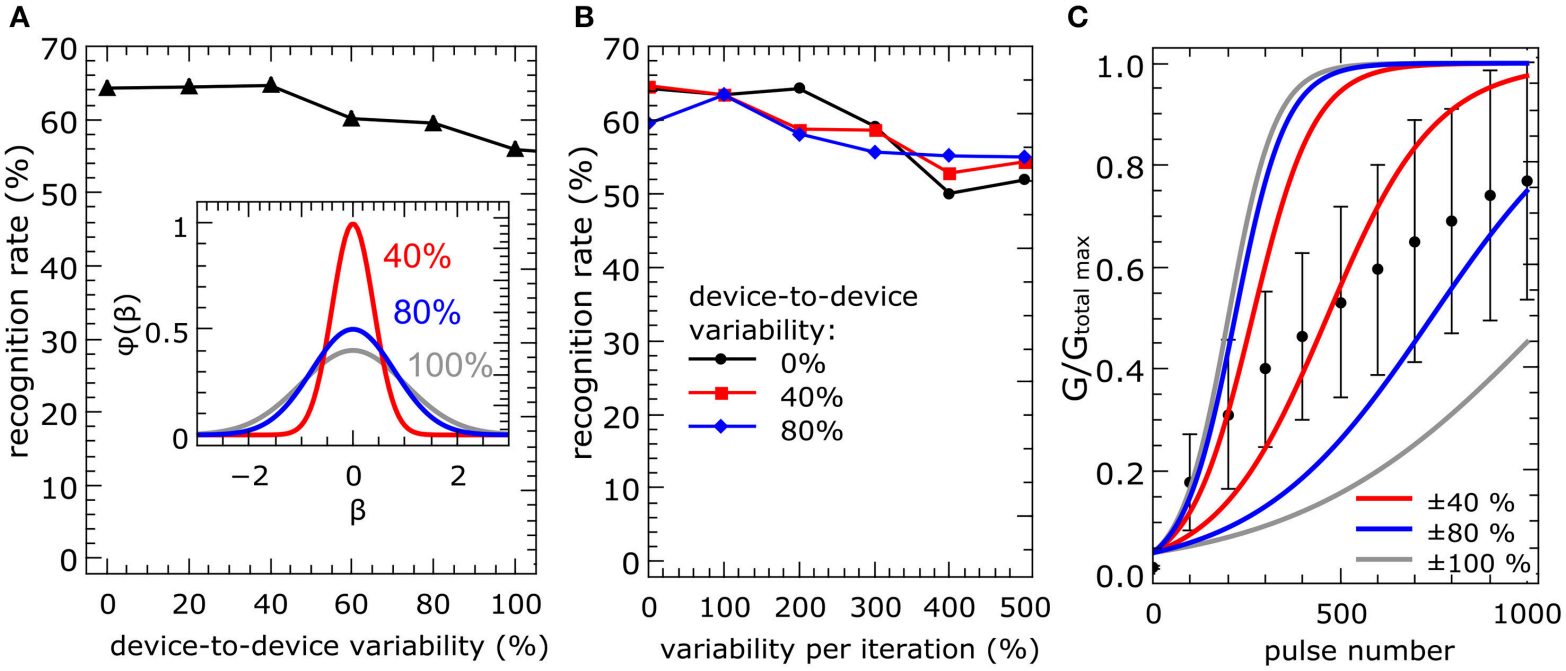
**Device variation: To test the reliability of the network model under device variation, the local learning rates of the memristive devices were changed to represent a Gaussian distribution φ(x)**. Each curve shows the obtained recognition rates for a full set of simulations for different standard deviations σ of the distribution function (see also inset in **A**). The presented data is averaged over three total simulation runs. In **(A)**, the local learning rate of every device was initially changed once, but kept constant over the simulation (σ_device_ is the standard deviation of the initial resistance state of the devices in the crossbar array). The learning rates for the simulation in **(B)** varied additionally in each iteration step for each device (σ_iteration_ is the standard deviation of the device resistance in each iteration step). In **(C)**, experimentally recorded data on the variation of the device resistance after every 100th potentiation pulse with amplitudes of 3.9 V and 1 ms length are compared with variations in the plasticity model for different σ_device_. The red, blue and gray lines indicate upper and lower boundaries, i.e., conductance evolution for the lowest and highest learning rate, for 40, 80, and 100% variability in conductance from device to device.

Three different scenarios have been investigated to determine the reliability, variability, and yield of the fabricated double-barrier memristive devices in the framework of the implemented network model: In the first scenario, a device-to-device variability was modeled. In this approach, the learning rates of all devices were initially changed once to represent a Gaussian distribution, but were kept constant during the simulation. The second scenario reflects the variability of individual memristive devices. In this case, the local learning rate of all memristive devices was varied in each iteration step. We also investigated a combination of the first and second scenarios, since this is the most likely case in reality. In all cases, the local learning rates β_*p*_ and β_*d*_ of the memristive devices (see Equation 3) were varied according to a Gaussian distribution.

(8) $$\varphi \left(x\right)=\frac{1}{\sqrt{\left(2\pi \sigma^{2}\right)}}\text{exp}\left(-\frac{x^{2}}{2\sigma^{2}}\right),$$

where $x=\beta_{p\left(d\right)}^{x}-\beta_{p\left(d\right)}^{0}$ (here β^*x*^ is the variable and β^0^ is the undisturbed learning rate defined in Equation 3) and σ denotes the standard deviation (see the inset of Figure 6A). The standard deviation of the device-to-device variability (first case) is denoted by σ_device_, while σ_iteration_ denotes the variability for each iteration step (second case). The simulation results are shown in Figure 6. The full set of 60,000 MNIST training images was applied three times to a network with 10 output neurons for the simulation. The presented data were averaged over three total simulation runs.

The recognition rates for the first scenario (increased device-to-device variability) are shown in Figure 6A. We found that, up to 50%, the recognition rate is not affected by the device-to-device variability and decreases only slightly with a further increase in device variability. The outcome of the second scenario is presented in Figure 6B. We found that the network is more robust under variations within individual devices (see the black line in Figure 6B). Even if we combine both, a device-to-device and individual device variability (see the red and blue curves in Figure 6B), a relatively robust network performance is obtained. Even if we combine both, a device-to-device and individual device variability (see the red and blue curves in Figure 6B), a relatively robust network performance is obtained. Therefore, a device-to-device variability up to 100% might be acceptable for a suitable network performance. Hence, our numerical investigation provides evidence that the most striking problems are introduced by a constant device-to-device variability. This can be explained by the fact that a variation in each iteration step is averaged over the total number of iteration steps. However, a constant variation of the local learning rate between the memristive devices, i.e., a device-to-device variability, leads to an ever-increasing variation with every iteration.

To estimate the performance of double-barrier devices within the network model considered here, it is necessary to analyze the experimentally obtained device variations in greater detail. Considering the error bars in Figure 3, the variation seems to be too high to fulfill the obtained network requirement of device-to-device variation of <100%. However, these error bars were determined from the final conductance after 1000 potentiation/depression pulses. A better representation of the device performance could be obtained by measuring the conductance variations after every 100th potentiation pulse. The obtained results are shown in Figure 6C. For a suitable comparison between simulated and measured data, the measured data were normalized by the highest recorded conductance *G*_max total_ (see Figure 6C). Furthermore, the solid lines in Figure 6C indicate the variation range of learning rates with, respectively, 40, 80, and 100% device-to-device variability. In particular, the experimentally recorded device-to-device variability is in the required variation interval. This suggests that the device variability can be assumed to be sufficient for the network learning process. Nevertheless, the device-to-device variability is one of the most challenging parameters for the realization of the pattern recognition computing scheme depicted in Figure 2.

**Figure 7:**
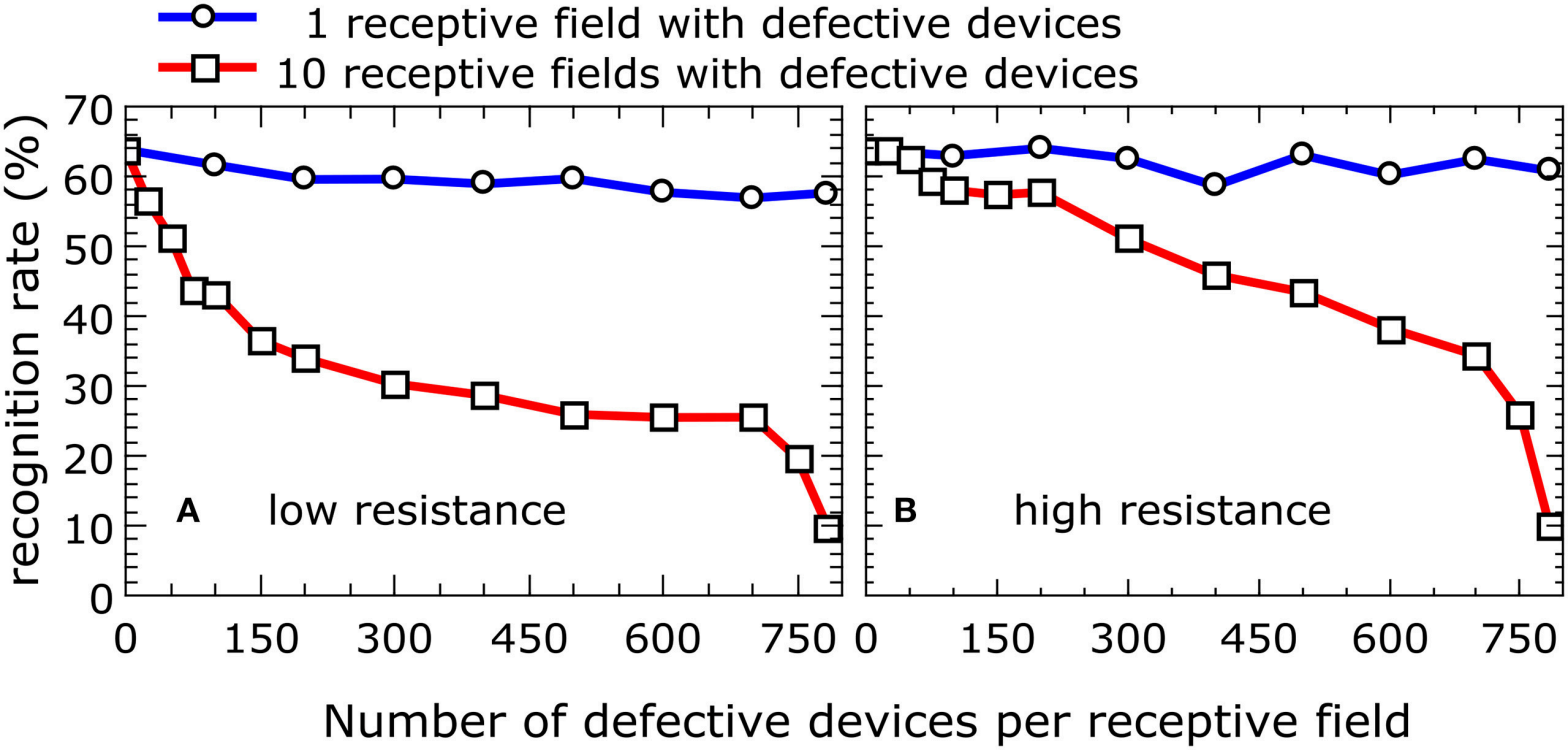
**Impact on the recognition rate of defective devices: Recognition rate as function of the number of defective devices with low and high resistance per receptive field**. The blue curve represents the situation where only one of the 10 receptive fields contains defective devices, while the red curve was obtained from a simulation where all 10 receptive fields contain defective devices. The full set of 60,000 MNIST training images was applied three times to a network with 10 output neurons for the simulation. The presented data were averaged over three total simulation runs. **(A)** Low resistance. **(B)** High resistance.

**Figure 8:**
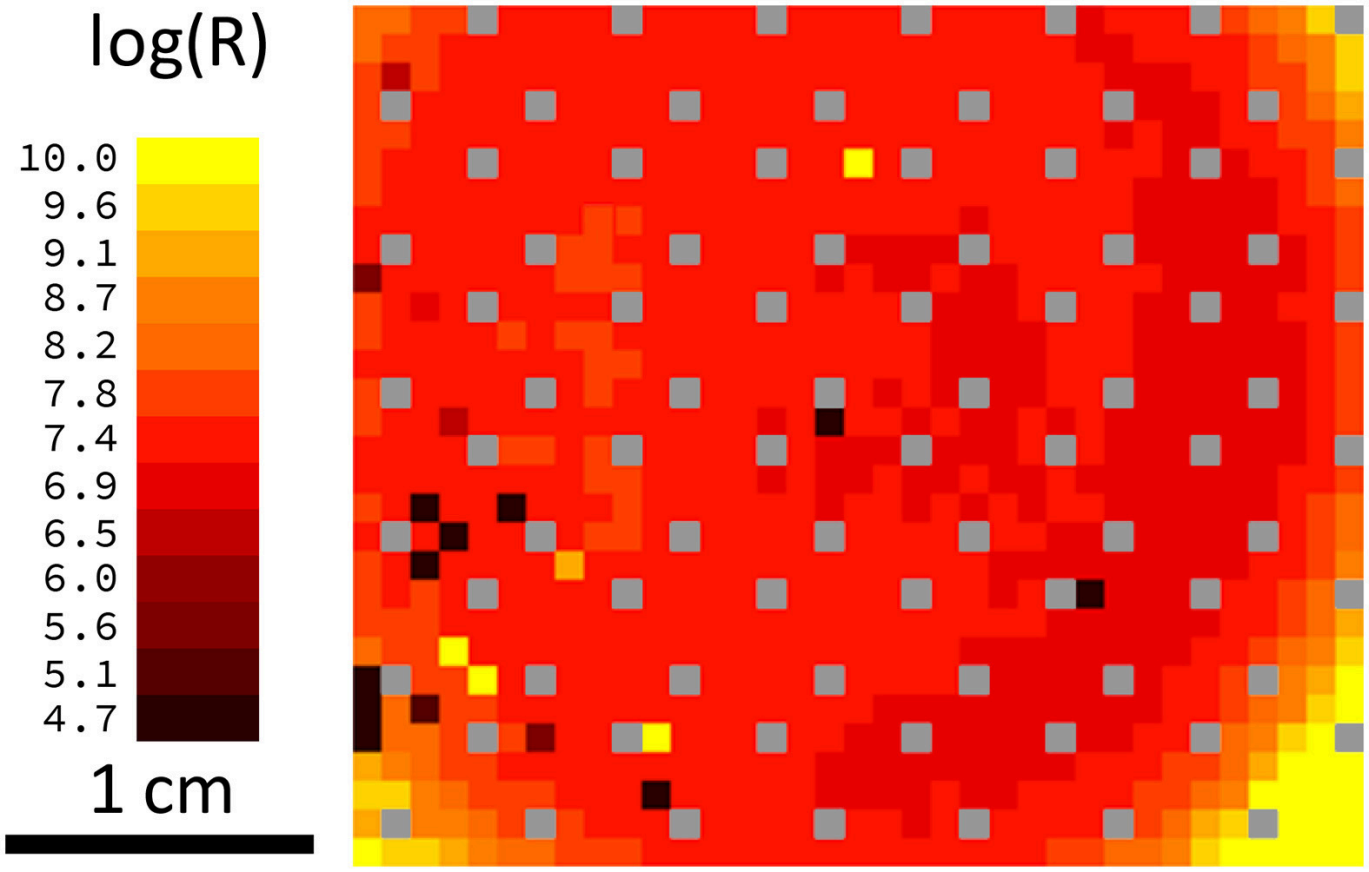
**Resistance distribution and yield: Wafermap showing the resistance of the memristive devices in the center of the wafer**. Each square represents one device with the color-coded resistance. Black squares are shorted devices; individual yellow squares are problems during the final metallization and gray squares contain no memristive devices (space used for testing structures). Yellow squares in the corners stem from different sputtering conditions at the border of the wafer. Considering the defective devices, the yield in the center of the wafer is ~98%.

Another possible negative impact on network performance can be introduced by defective devices. Two scenarios can be distinguished here: devices with a low resistance (e.g., devices that are shorted) and devices with a high resistance (e.g., those that are not properly connected during the final metallization step). In either case, during the simulation, the resistance was assumed to be constant. The most problematic situation arises from devices that are initially in the low-resistance state and are not able to perform resistive switching under voltage pulsing. Owing to the increased conductivity of the device and the subsequent greater weight during the training phase, the receptive field for a given column of devices will be distorted. To study the influence of such devices on overall network performance, the dependence of the recognition rates on the number of defective devices was calculated. The results for a network with 10 output neurons are shown in Figure 7A, where the blue curve represents the situation where only one of the 10 receptive fields contains defective devices, while the red curve was obtained from a simulation where all 10 receptive fields contain defective devices. If only one receptive field contains defective devices, the overall recognition rate of the circuit is nearly constant up to 784 defective devices. However, if all 10 receptive fields above the output neurons contain defective devices, the recognition rate is drastically decreased. For defective devices with an unchangeable high resistance, the recognition rate shows significantly different behavior (Figure 7B). In comparison with defective low-resistance devices, defects in high-resistance devices do not drastically change the formation of the receptive fields. However, the observed influence of defective devices on network performance can be assumed to play a minor role owing to the high quality of the fabrication process. Figure 8 shows a map of a typical recorded resistance distribution across a wafer. We should mention here that only the center of the 4-inch wafer is depicted, because the 4-inch sputter targets limit the usable area on these wafers. Each square represents the resistance of one memristive device (although the actual density of devices is larger because only one in six devices was measured). In this wafer map, a total of 966 devices were measured. Besides the working devices, 14 devices (black squares) are defective in terms of an initial low resistance (i.e., are shorted), while five devices (yellow squares) have an unexpected high resistance (e.g., as a result of problems with the final metallization step). Yellow squares at borders (e.g., the lower right corner) are devices with a significantly larger resistance, which can be attributed to side effects of the sputtering process. In the interesting center region, the yield is thus about 98%, which is typical for our wafers. Comparing the number of defective devices with Figure 7, it is obvious that the recognition rates will remain nearly unaffected.

In conclusion, we have provided evidence that double-barrier devices are interesting candidates for use as artificial synapses in neuromorphic circuits. In particular, the gradual change in their resistance under voltage pulsing and the resulting reliability, variability, and yield of such devices might fulfill the requirements of neural networks. An experimental realization of the use of these components seems possible and may pave the way to a real-time implementation of a pattern recognition system.

## Author contributions

MH prepared the samples, performed the measurements, analyzed the experimental results and co-wrote the manuscript. MZ supported the measurements and data interpretation. FZ and MZ developed the simulation model. The simulation results were discussed and interpreted between MZ, FZ, MH, and HK. HK and MZ conceived the idea, initiated, and supervised the experimental research. MH, MZ, and HK discussed the experimental results and contributed to the refinement of the manuscript.

### Conflict of interest statement

The authors declare that the research was conducted in the absence of any commercial or financial relationships that could be construed as a potential conflict of interest.
